# Making prescriptions “talk” to stroke and heart attack survivors to improve adherence: Results of a randomized clinical trial (The Talking Rx Study)

**DOI:** 10.1371/journal.pone.0197671

**Published:** 2018-12-20

**Authors:** Ayeesha Kamran Kamal, Wardah Khalid, Abdul Muqeet, Anum Jamil, Kashfa Farhat, Sehar Rahim Ali Gillani, Maryam Zulfiqar, Mehreen Saif, Aliya Amin Muhammad, Fabiha Zaidi, Mohammad Mustafa, Ambreen Gowani, Shahrukh Sharif, Syedah Saira Bokhari, Javed Tai, Nasir Rahman, Fateh Ali Tipoo Sultan, Saleem Sayani, Salim S. Virani

**Affiliations:** 1 Section of Neurology, Department of Medicine, Aga Khan University, Karachi, Pakistan; 2 Stroke Service, Department of Medicine, Aga Khan University, Karachi, Pakistan; 3 Aga Khan Development Network e-Health Resource Centre, Karachi, Pakistan; 4 Stroke Services, Aga Khan University, Karachi, Pakistan; 5 Stroke Service, Aga Khan University Hospital, Karachi, Pakistan; 6 Aga Khan Development Network Health Resource Centre, Karachi, Pakistan; 7 School of Nursing & Midwifery, Aga Khan University, Karachi, Pakistan; 8 Aga Khan Development Network Health Resource Centre, Karachi, Pakistan; 9 Section of Cardiology, Aga Khan University, Karachi, Pakistan; 10 Section of Cardiology Department of Medicine, Aga Khan University, Karachi, Pakistan; 11 Aga Khan Development Network e-Health Resource Centre, Karachi, Pakistan; 12 Michael E. DeBakey Veterans Affairs Medical Center & Section of Cardiovascular Research, Department of Medicine, Baylor College of Medicine, Houston, Texas, United States of America; IRCCS E. Medea, ITALY

## Abstract

**Background:**

We developed and tested the effectiveness of a tailored health information technology driven intervention: “Talking Prescriptions” (Talking *Rx*) to improve medication adherence in a resource challenged environment.

**Methods:**

We conducted a parallel, randomized, controlled, assessor-blinded trial at the Aga Khan University (AKU), Karachi, Pakistan. Adults with diagnosis of cerebrovascular accident (CVA) or coronary artery disease (CAD) diagnosed least one month before enrollment, on anti-platelets and statins, with access to a mobile phone were enrolled. The intervention group received a) Daily Interactive Voice Response (IVR) call services regarding specific statin and antiplatelet b) Daily tailored medication reminders for statin and antiplatelet and c) Weekly lifestyle modification messages for a period of 3 months. We assessed Medication adherence to statin and antiplatelets by a validated version of the 8-item Morisky Medication Adherence scale 8 (MMAS-8) at 3 months by a blinded assessment officer. Analysis was conducted by intention-to-treat principle (ITT).

**Results:**

Between April 2015 and December 2015, 197 participants (99 in intervention and 98 in the usual care group) enrolled in the Talking Rx Study. The dropout rate was 9.6%. Baseline group characteristics were similar. At baseline, the mean MMAS-8 was 6.68 (SD = 1.28) in the intervention group and 6.77 (SD = 1.36) in usual care group. At end of follow-up, the mean MMAS-8 increased to 7.41(0.78) in the intervention group compared with 7.38 (0.99) in usual care group with mean difference of 0.03 (S.D 0.13) (95% C.I [-0.23, 0.29]), which was not statistically significant. (*P-Value* = 0.40) CVA patients showed a relatively greater magnitude of adherence via the MMAS-8 at the end of follow up where the mean MMAS-8 increased to 7.29 (S.D 0.82) in the intervention group as compared to 7.07(S.D 1.24) in usual care group with mean difference of 0.22 (SD = 0.22) 95% C.I (-0.20, 0.65) with (*P*-value = 0.15). Around 84% of those on intervention arm used the service, calling at least 3 times and listening to their prescriptions for an average of 8 minutes. No user was excluded due to technologic reasons.

**Conclusion:**

The use of a phone based medication adherence program was feasible in LMIC settings with high volume clinics and low patient literacy. In this early study, with limited follow up, the program did not achieve any statistically significant differences in adherence behavior as self—reported by the MMAS-8 Scale.

**Trial registration:**

Clinical Trials.gov NCT02354040.

## Introduction

Cardiovascular diseases (CVDs) namely cerebrovascular accidents (CVA) and or coronary artery disease (CAD) are leading causes of death globally. An estimated 17.5 million people died from CVDs in 2012, representing 31% of global deaths. Of these deaths, an estimated 7.4 million were due to CAD and 6.7 million were due to CVA. Over three quarters of CVD related deaths take place in low- and middle-income countries. [1]

Pakistan is the sixth most populous country in the world. It has a reported lifetime prevalence of stroke symptoms of 19% in the transitional urban population. [2, 3, 4] One in four of middle-aged adults in Pakistan suffers from CAD. [5] Thus, like other emerging LMIC countries, it bears a disproportionately large burden of vascular disease. Life style modification, medical risk identification and control via adherence to medications are established cost effective strategies for secondary prevention. [6] Medications alone can reduce the risk of stroke by 30% and of Myocardial Infarction (MI) by 15%. [7] However, adherence, even in developed countries is around 50%. [8] In Pakistan, reported adherence to cardiac medicines ranges from 27–77% and about 68% to stroke medicines in the first 2 years after event. [9,10]

There is considerable room for improvement to increase adherence in population at risk both in Lower Middle Income Countries (LMIC) and in Higher Income Countries (HIC) settings. However, interventions to improve compliance like drug dairies, pill counters, automated reminders and social support would not be feasible in a resource poor, literacy challenged LMIC setting. [11] Due to an out of pocket, self-referral system, patients frequently take multiple opinions and prescriptions from different physicians at the same time. [12,13] This leads to increased risk for drug-drug interactions and a decline in adherence behavior.

m-Health based strategies have the potential to be an inexpensive and easily accessible tool to increase compliance and bridge the communication gap between health care providers and users. [14] Cellular phone technologies have been used in the past to increase medication adherence in HIV [15], smoking cessation [16], diabetes [17], maternal and child health care. [18] According to the Pakistan Telecommunication Authority, the total cellular density in Pakistan is reported to be 77% and 92% of Pakistan has internet coverage. [19] Leveraging this fact, we developed and pilot tested a patient individualized tailored health information technology-driven intervention in Pakistani patients with CAD and CVA receiving care in the outpatient setting of a busy tertiary care hospital. We hypothesized that IT based education via interactive Talking Prescriptions would lead to an increase in medication adherence due to the improved health literacy provided by talking prescriptions compared to usual care.

## Methods and material

### Trial design

The Talking *Rx* trial was a parallel-design, assessor-blinded, randomized controlled single center trial enrolling patients suffering from CAD or CVA. The Talking Rx study was conducted from April until December 2015. Research team members evaluating the participants at the end of follow-up period were blinded to the assignment. The participants were randomized into two parallel groups in a 1:1 ratio via block technique with usual care group receiving the standard of care as per institutional guidelines while the parallel intervention group also received Talking Rx intervention based on: 1) Daily Interactive Voice Response (IVR) call services providing information about specific statin and antiplatelet, 2) Daily tailored medication reminders for patients to take their statin and antiplatelet medication and 3) Weekly Life Style Modification Messages that engaged the patient and were also related to medication adherence. We chose statins and antiplatelet medications as the focus of our intervention as these medications have been shown to decrease the risk of recurrent cardiovascular events in patients with CAD and CVA. Following is a brief outline of the methodology used in the study. For further details, please refer to the published protocol. [20]

### Study setting

The Talking *Rx* trial was conducted at the Aga Khan University Hospital (AKUH) in Karachi, Pakistan. All the patients admitting with CAD or CVA events receive standard of care treatment according to International and institutional guidelines. The Talking *Rx* trail procedures were performed at Clinical Trial Unit (CTU) of AKUH, which included assessment of eligibility, baseline assessment, process of randomization and administration of intervention and follow-up visit.

### Study participants

Potential participants were identified through daily screening of cardiology and neurology outpatient clinic lists. Patients with history of CVA or CAD attending neurology and cardiology clinics at AKUH, who fulfilled the study eligibility criteria and could provide written informed consent, were recruited as study participants based on the following criteria:

### Eligibility criteria

#### Inclusion criteria

- Age older than 18 years both male and female gender
- History of CVA or CAD [(MI, unstable angina, or coronary intervention performed via percutaneous coronary intervention or coronary artery bypass grafting (CABG)]. These were confirmed using objective modalities at the time of the episode (i.e., neuroimaging, electrocardiogram (ECG), cardiac catheterization, relevant blood tests, physician examination and clinical confirmation in medical records).
- More than 1 month since last episode of CVA or CAD.
- Use of anti-platelets and statins both.
- Modified Rankin Score of 3 or less. (for CVA patients)
- Possession of a mobile phone that the patient has access to at all times. In the case of patients who did not own a mobile phone or were unable to use a mobile phone, they were enrolled if they had a primary caregiver available at all times who owned a cell phone.
- Ability to receive and comprehend an SMS (Short Message Service) in English or Urdu. In the case of patients who themselves were unable to receive or comprehend an SMS, they needed to have a caregiver available at all time who could perform the above-mentioned tasks.

#### Exclusion criteria

- Planned travel within 2 months of recruitment in an area where phone internet coverage is known to be underdeveloped or not present.
- History of malignancy (diagnosed in the last 5 years and/or receiving active treatment) requiring medication adjustment.
- Planned procedure (during the study time period of 3 months) which necessitates rapid medication changes like CEA (Carotid Endarterectomy), CABG, or percutaneous coronary intervention).

### Randomization

The Clinical Trial Unit staff, separate administrative entities from the research staff at AKUH performed randomization. Centrally Randomized computer generated random allocation sequence was used by the CTU. For allocation concealment, opaque white envelopes were used to ensure that the next assignment was not predictable. Participants were assigned to the two groups in a parallel fashion in a 1:1 ratio. Block randomization technique with a block size of 10 was used to make the next assignment unpredictable to the research team who were not aware of block sizes.

### Intervention group

**Fig 1:**
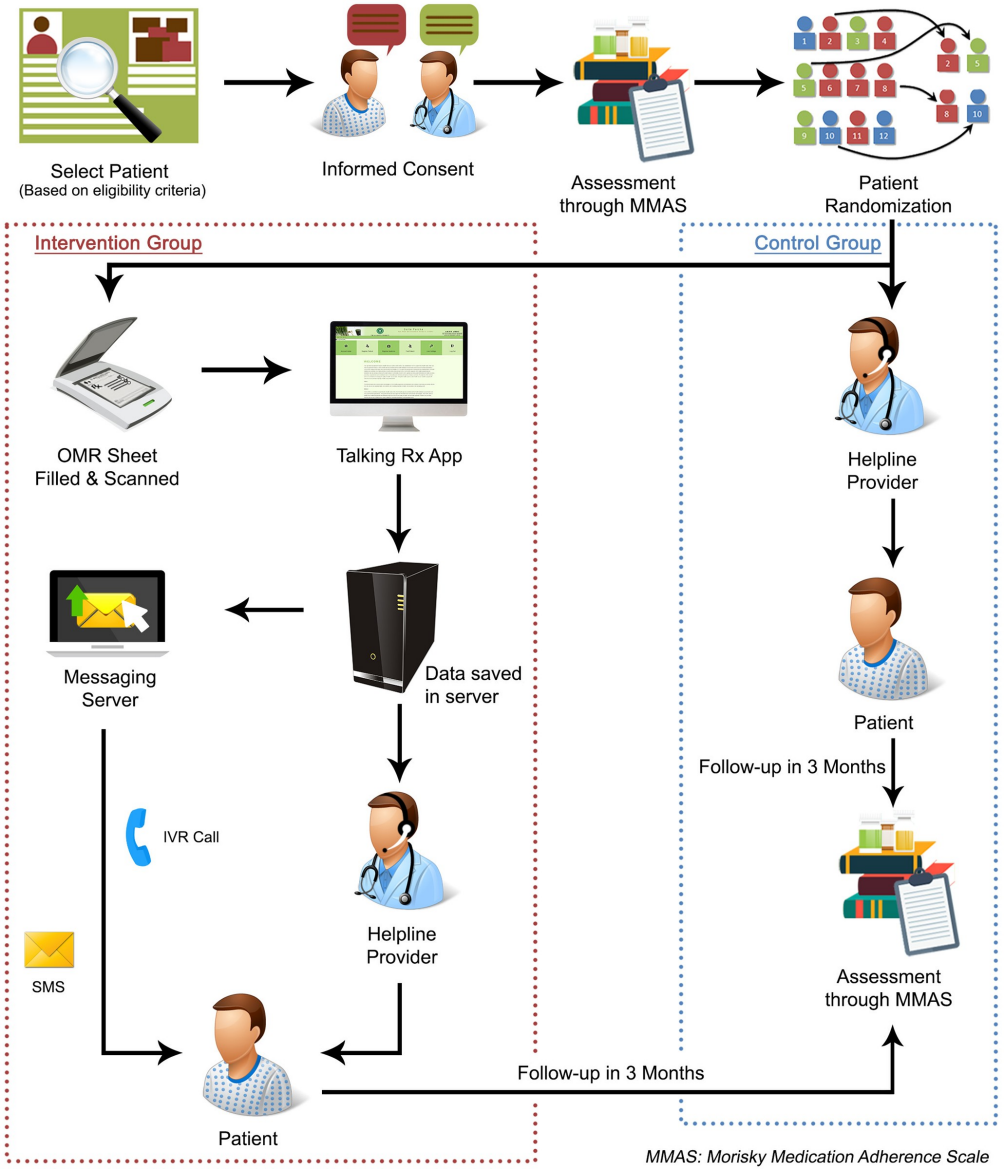
Study activity flow diagram.

**Fig 2:**
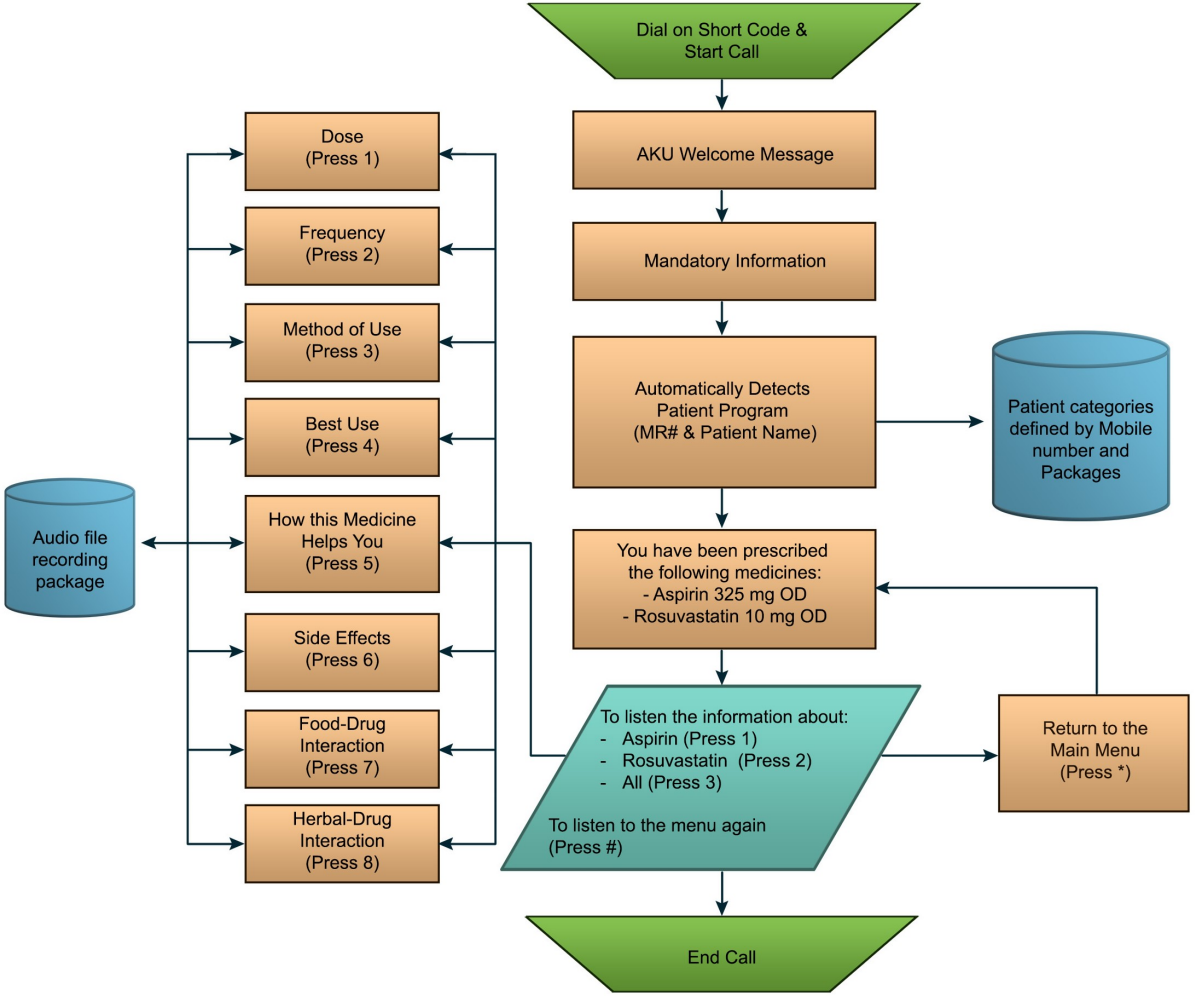
Flow diagram of Interactive Voice Response (IVR).

Participants in the intervention group received daily Interactive Voice Response (IVR) call services, daily prescription tailored medication reminders and once weekly life style modification messages. The IVR service was tailored to each patient prescription for anti-platelets or statin. (“Fig 1 Study Activity Flow Diagram”) The intervention focused on two sets of medications (anti-platelets and statins) widely used in the treatment of patients with CVA and CAD. The information on IVR was available on four sets of generic anti-platelet medication, which included Aspirin, Clopidogrel, Cilostazol and Prasugrel. The four sets of generic statins included Atorvastatin, Rosuvastatin, Simvastatin and Ezetimibe. After randomization, participants were registered in the Talking *Rx* software system. This software was linked to a central program. Optical Mark Recognition Sheet (OMR sheet) was used to mark patient’s prescription. The marked OMR sheet was then scanned into the Talking *Rx* software to directly enter patients tailored prescription in the software. Once the software received the prescription, the data was transferred to the central program of IVR and participants received a welcome test message which showed successful activation of IVR services tailored to patient’s prescription. The welcome message contained a number and by dialing that number, participants were able to receive IVR services. Once the participants made a free call on the specific number, the call was registered on the central system and they received a call back where the IVR was played. First, a mandatory message was played explaining the important instructions pertaining to all medicines followed by the recording of their specific medicines. The participants had the choice to listen to full recording or select different options listed on the IVR Menu. They could listen to their strength (dose) of medicine, its prescribed frequency, how and the best time to take the medicine, drug-drug and drug-food interactions and side effects. (“Fig 2 Flow diagram of Interactive Voice Response (IVR)”) The participants had a choice to listen to each subcategory serially, or targeted information as desired, once or multiple times. This service was available free-of-cost 24/7. This modality focused on educating the patient regarding basic mediation related literacy. It also addressed the common myths that surround medications in health literacy challenged areas e.g., the myth that one can feel chronic disease symptoms, and therefore, can stop taking medications by themselves. The content of the IVR was systematically developed by a qualified pharmacist and clinical team, based on evidence-based guidelines and recommendations and input by patients who had survived CVA and CAD via iterative qualitative assessments. The content was reviewed and expert validated by a team of stroke neurologist and cardiologist to authenticate its relevance and clarity.

**Fig 3:**
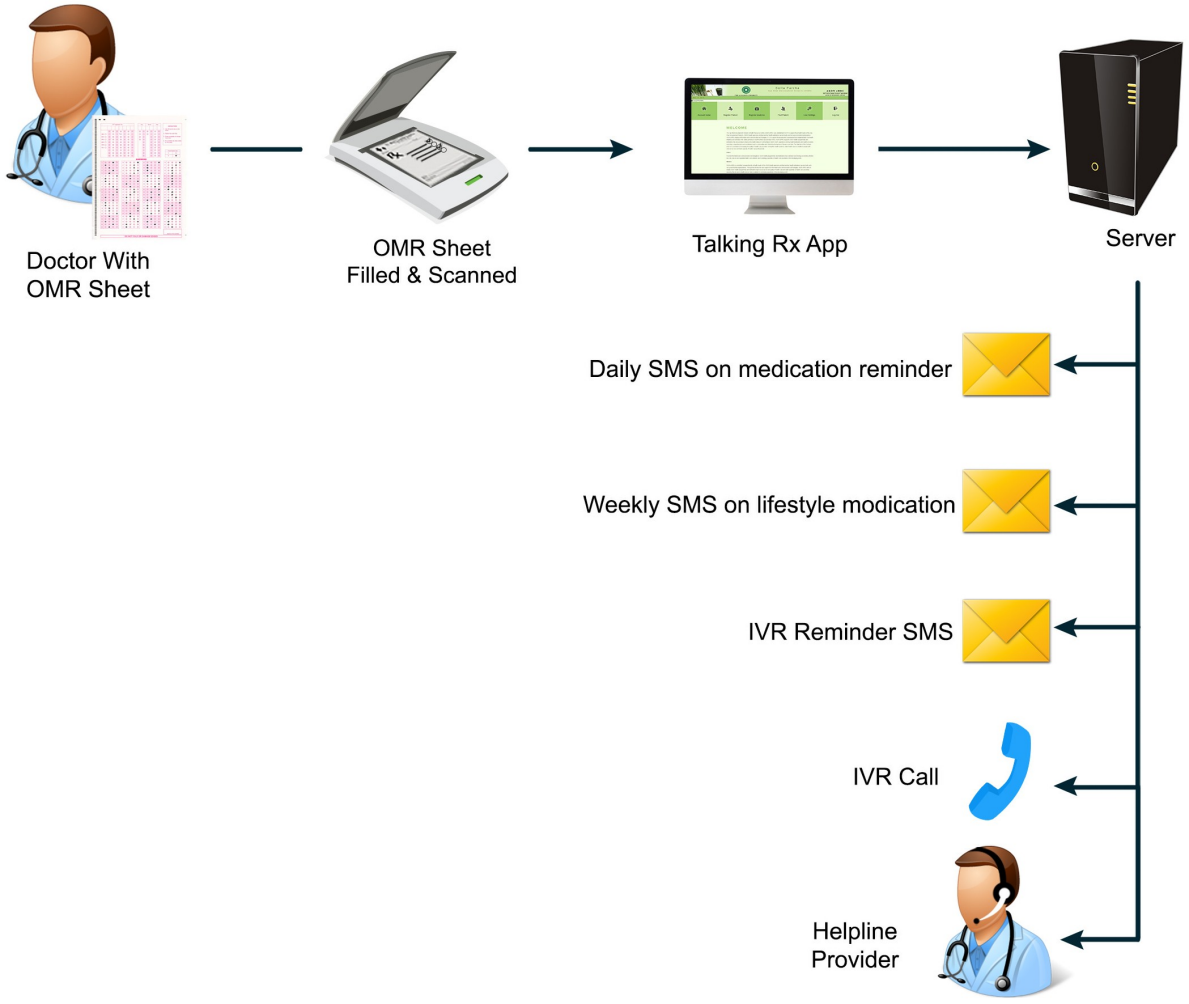
Experience of users in the intervention group.

**Fig 4:**
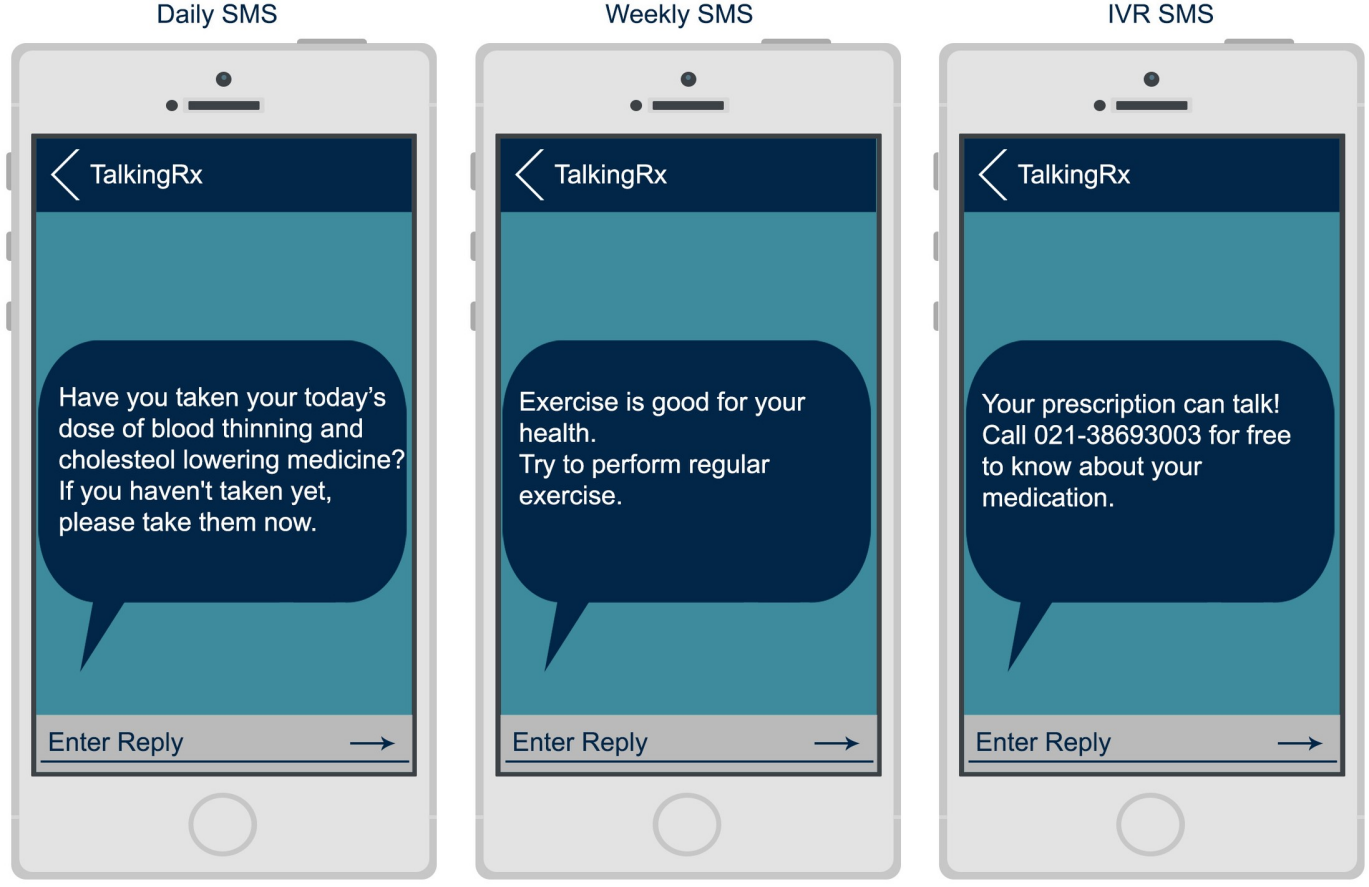
Screen shots of IVR and SMS reminders messages.

In addition, participants also received a daily-automated SMS highly personalized to their anti-platelet and statin prescription to remind them to take their medication. This SMS was sent 5 days a week in the early afternoon, which was identified as the most convenient time by the participants to receive reminder messages. Furthermore, one life style modification SMS was sent only once weekly to reinforce the importance of medication adherence, lifestyle modifications and cardiovascular risk factors. (“Fig 3 Experience of Users in the Intervention Group, Fig 4 Screen shots of IVR and SMS Reminders messages”) The intervention was aimed to boost medication adherence and medication health literacy. The reminder SMS were phrased using the behavior change intervention taxonomy devised by Michie et al. [21] The specific content of the SMS was derived from the Social Cognitive Theory and Health Belief Model of behavior change in health care. [22]

### The Talking Rx program

The Talking *Rx* program was an IVR technology based program specifically designed by the Aga Khan Development Network eHealth Resource Centre (AKDN eHRC) in collaboration with the clinical team that worked in these high volume clinics. The application was customized for the program. It was not an open source system. The hardware and software details for the program are as follows:

#### Hardware

The hardware used in the intervention is as follows:

- Laptop—A laptop was used to install the Talking Rx application, which was developed in-house. Multiple users were able to login the application but one at a time. This laptop also acted as a local server.
- Scanner—A scanner was connected with the laptop for scanning the Optical Mark Recognition (OMR) sheet where optical mark recognition is the process of capturing human-marked data from a document.

#### Software

In order to develop the customized application we used the following software:

- PHP (server-side scripting language)–It is a server scripting language, and a powerful tool for making dynamic and interactive web pages.
- SQL (Structured Query Language)–It is a domain-specific language used in programming and designed for managing data held in a relational database management system, or for stream processing in a relational data stream management system.
- Java—It is a general-purpose computer programming language that is concurrent, class-based, object-oriented, and specifically designed to have as few implementation dependencies as possible.
- MATLAB (Matrix Laboratory)–It is a multi-paradigm numerical computing environment. MATLAB allows matrix manipulations, plotting of functions and data, implementation of algorithms, and creation of user interfaces.
- XML (eXtensible Markup Language)–It is designed to store and transport data that is both human- and machine-readable.

#### Desktop-based imaging application

It was used for scanning the prescription page, detecting markers of medications, dose and frequency and translating into an XML form which was understandable by the web-based medical record application.

#### Web-based medical record application

This was a web-based application that was locally hosted, which registered the patient, saved patient’s pharmacy data, translated XML into record form and communicated with other messaging server through CSV files and HTTP protocols.

This application sent CSV file to voice server using internet services. In addition to the above mentioned applications, the system had a messaging and voice server. This server was hosted at the service provider and contained an application which stored all audio files in coded form (encrypted). The application accepts CSV file format from the web-based server and binds specific audio files and messages based on the prescribed medicines with patient’s mobile phone number. This server allows web-based medical record to send SMS to patients through HTTP protocols and patients can call the server from their mobile phones to hear their prescription “talk”.

**Audio files**:

The audio files were developed in house by a team of pharmacists, physicians and content experts who reviewed the script, and they were recorded and reviewed with the support of the i n—house Audio Visual Department and the eHealth resource centre where the studio was provided. Prior to deployment, the content was played back and checked for inaccuracies and finalized and edited recordings were added to the program.

There were two types of audio recordings:

*General*: This contains general messages recordings such as welcome message and thank you message at the beginning and at the end of each call respectively; the doses of the medicines such as 5mg, 10mg, 20mg etc.; the frequency of the medicines such as morning, evening, night, before meal, after meal etc.
*Specific*: This contains medicine specific messages such as benefits, side-effects, contraindications, method of use of each medicine.

The third party application managed the audio recordings and played the recordings as per the prescribed medicines to the patient. So when a patient calls, they hear only their prescribed medicine details.

#### Technical flow

**Fig 5:**
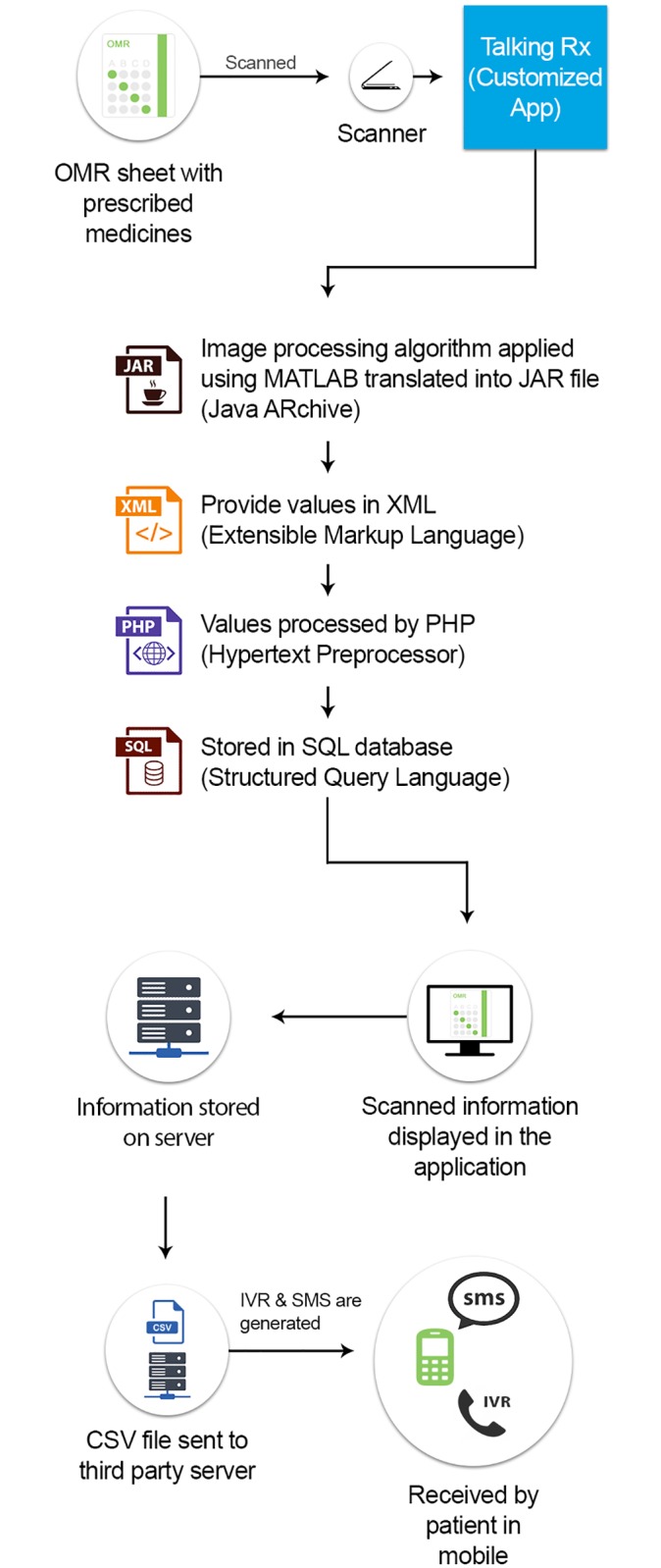
Technical details of intervention flow for further clarity.

The technical flow of the intervention is as follows (Please refer to “Fig 5 Technical Details of Intervention Flow for further clarity”). Firstly, Patient Registration: The patients were enrolled in the application by entering their demographic information such as name, age, gender etc. The next step was, Acquistion and Processing: The medicines were prescribed on an OMR sheet by shading in the area of the relevant medicine for the patient. These OMR sheets were scanned by the scanner and shaded areas were detected by the image processing algorithm developed using the MATLAB. This algorithm was then translated into Java readable file i.e. JAR file. This JAR file passes the image through image processing algorithm and provides values in XML. The detected values of the image processed, provides medication name and other information that can be extracted from XML script using PHP coding. PHP program can use Java script to use JAR file in web environment and extracted data from the image can be stored in SQL database. The XML file is used to communicate with other messaging server through Comma-separated Values (CSV) files and Hypertext Transfer Protocol (HTTP). The OMR sheet, MATLAB, XML and PHP/Javascript are only necessary to get the patient and prescription information into an SQL database so that the Talking Rx program can then be activated in an electronic medical record system, this step may be skipped in favour of a digital system and were necessary due to local context rather than the principle of the system.

**Fig 6:**
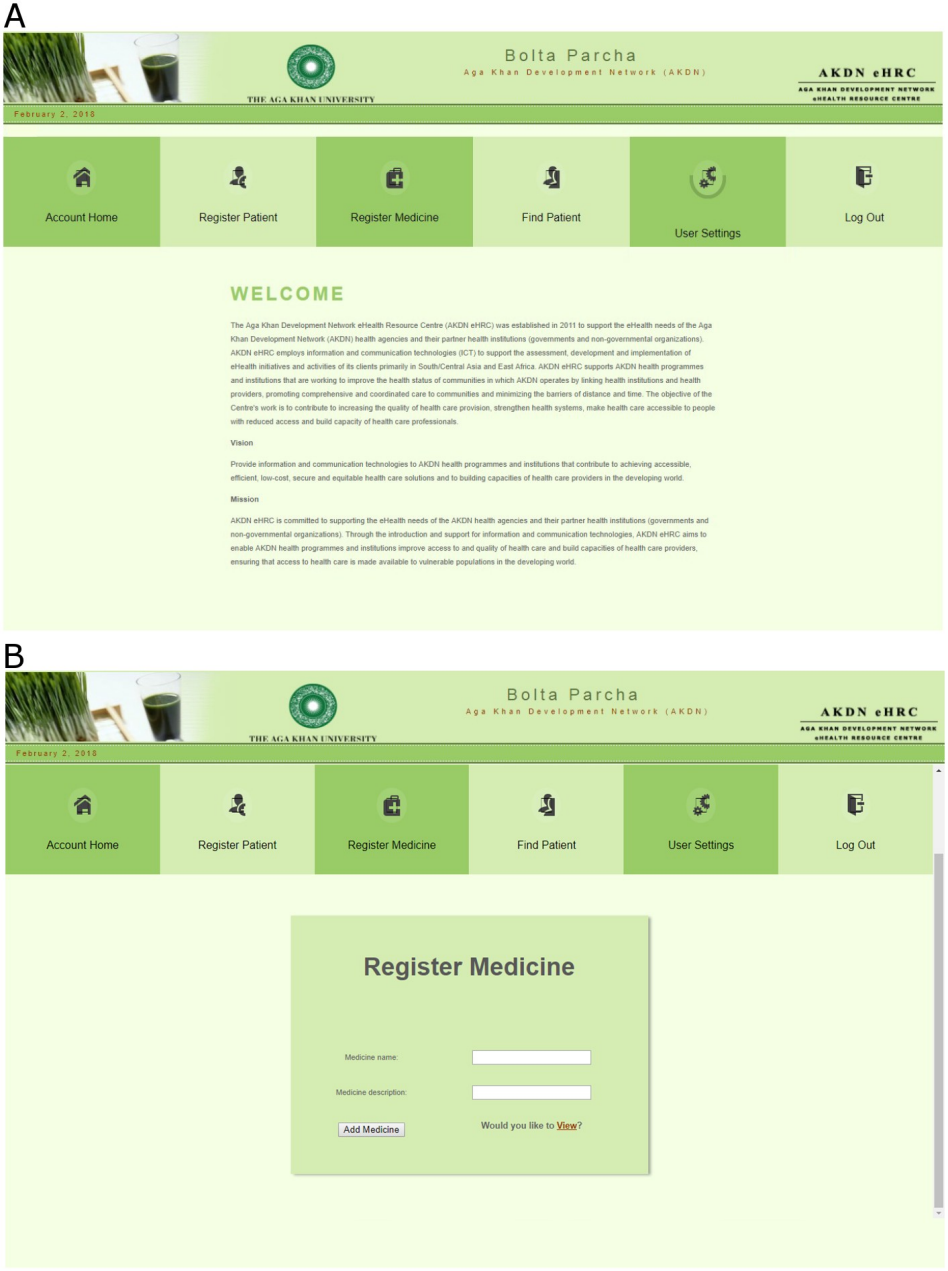
6a and 6b. Screen shot web portal.

The CSV file was sent from the application to the third party server for the SMS and Interactive Voice Response (IVR) services. This server was hosted at the service provider and contained an application which stored all encrypted audio files. The CSV file containing patient information regarding demographics and medicines binds with the prescribed medicine-based audio recordings and messages. The purpose of the messaging and server was to send SMS, related to patient education and behaviour change, to patients through HTTP along with IVR number. The system had a Voice Response capacity, the patients called the server from their mobile phones using the provided IVR number to hear their prescription “talk”. The call provides a welcome message and information on the prescribed medicines such as dose, frequency, method of use, side effects, herbal-drug interaction etc. Based on diseases and prescribed drugs, the web-based system sends customized messages to patients through messaging sever periodically. Through this feature, behaviour change communication is established to promote positive health related behaviour in patients. The data was monitored on the web portal. The data is exportable and reviewable, providing user interface interaction data. (“Fig 6a and 6b Screen shot Web Portal”)

### Usual care group

In the usual care group, participants received the usual standard of care provided for CVA and CAD patients. This primarily consists of regular follow-up visits (frequency as per patient needs) with their stroke neurologist or cardiologist.

### Study helpline

In addition, every participant whether in the intervention or the usual care group also received access to a helpline number where they could contact the research team during the study duration. Participants could post any queries pertaining to their illness or questions related to their medications on this helpline number. These queries were answered within 24 hours by a study physician.

### Study procedures

Participants who fulfilled the eligibility criteria and provided written informed consent were invited to the Clinical Trials Unit. All participants were assessed at baseline for data on socio-demographic information, medical and prescription details. The baseline adherence score for each participant was evaluated by the Urdu version of the 8-item Morisky Medication Adherence Scale 8(MMAS-8- Urdu Version) [23, 24] followed by randomization to either the intervention group or the usual care group. After allocation, the research staff explained the details of the intervention to the participants. The participants were registered on the Talking *Rx* program. The study team also demonstrated how to use the system by sending one test SMS on participant’s mobile phone. In case of allocation to usual care group, the participants were informed about their dates of follow-up at the end of 3 months. The study research staff (medical doctors and pharmacist) was divided into two groups. Those who randomized and administered the intervention were separate from those who performed assessment of study outcomes. Those performing outcome assessment were geographically separate from the randomization and intervention deployment team.

### Follow-up and outcome ascertainment

Follow ups were performed at 3 months post intervention vs. usual care. In order to minimize the dropout rate, participants in both groups were reminded one week prior to their planned follow-up visit. Two day prior to visit, they were reminded through a SMS. If the participants were not able to come for their follow-up at 3 months, a period of +14 days was permitted to maximize follow-up. Participants, who were not able to come to CTU for follow-up because they were not in the city, were contacted and their follow-up was performed on the telephone. Outcome assessment was performed by trained study research staff who were blinded to the assignments of participants.

### Primary outcome measure

The outcome of interest was a change in medication adherence after 3 months of receiving the Talking *Rx* intervention. Medication adherence was assessed at baseline and after 3 months in both groups by administrating the Urdu version of the 8-item Morisky Medication Adherence Scale 8 (MMAS-8 Urdu). [23, 24] The scale has been translated and validated in Urdu and has a sensitivity of 46% and specificity of 60%. [24] The scale consists of eight questions. The maximum score is 8, score of < 6 denotes low adherence, 6 to <8 denotes medium adherence and a score of 8 denotes high adherence. This study has used the Urdu validated scale of MMAS-8.

### Process measures: Technical monitoring, recording deployment fidelity and uptake

Continuous monitoring of data was performed by the technology team at AKDN eHRC to assess the fidelity to the intended intervention during deployment by the operators and uptake of the intervention by the users. We were able to record how many participants accessed and used the intervention and what parts of the talking prescription were used repeatedly. The digital server was also recording fidelity of deployment in that the data was flowing without hitches as intended, monitoring access and interoperability issues due to separate phones and systems accessing the program. Data was collected on technology indicators like the total numbers of calls made on IVR and the average duration of IVR recordings.

### Sample size calculation

The sample size was calculated to detect a difference of 1 point difference (SD = 2) on the primary outcome measure (MMAS-8) between the two groups. Based on these assumptions, the estimated sample size requirement for the study was 86 participants in each group required to achieve a power of 90% and significance level of 5% when testing a two-tailed hypothesis of inequality of means. Accounting for a 15% attrition rate, the required sample size required for the study was 100 participants in each group, making total of 200 participants to be randomized. The MMAS-8 categorizes low adherence as a score of <6 medium adherence as a score of 6 to <8, and high adherence as a score of 8. [24] Any 1-point shift is a clinically important increase in medication adherence. Locally carried out studies have shown the effect of IT-based interventions on this score in these ranges. [17, 25]

### Statistical analysis

The analysis was performed based on the intention-to-treat principle. Analysis was performed after the completion of follow-up for all study participants. For descriptive analysis, mean with standard deviation or median with Interquartile Range (IQR) are reported based on normality assumptions for continuous data. For categorical data, frequency with percentages are reported. The primary outcome analysis compared the improvement in medication adherence level, assessed by the change in MMAS-8 score at 3 months of follow-up between the two groups. For these analyses, MMAS-8 score was used as continuous variable. Independent t test for two samples was applied to report the difference between the mean MMAS-8 scores for the intervention and the usual care group. Further sub-group analyses were performed to study the effect of intervention on CVA and CAD participants. Finally, we performed User interface experience analysis to report the feasibility and the uptake of the intervention by the study participants. [26] All analysis was performed on Stata version13.

### Acceptability of intervention by participants

At the end of follow-up visit, participants were asked to share their opinions, experience and recommendation on how to further improve the intervention. At exit follow-up interview, we asked open-ended questions for end user feedback. All of the participants were asked to respond to questions like what was your experience while using the intervention? What further changes can be brought to the intervention? and share any practical experiences and opinions regarding the intervention? In the end the feedback received, was analyzed by manual content analysis to identify the relevant thematic findings obtained thorough qualitative based open-ended questions.

### Ethical review and patients confidentiality

The study protocol was approved by the Aga Khan University Ethical Review Committee (ERC) with reference no 3165-MED-ERC_14. The study was also registered online at the clinical trails.gov website with ClinicalTrials.gov Identifier NCT02354040. Research team was trained and certified in Ethics and Good Clinical Practice (GCP) certification. The team received one-week training before the start of the study. This training included study design, patient confidentiality and ethical issues. Strict confidentiality and privacy was ensured.

All participants provided written informed consent at the time of recruitment which was available in both English and Urdu. Participants had the right to withdraw from the study at any time. If the stroke patient was not able to sign the informed consent due to their disability, then consent was taken from their caregiver in the presence of a witness. Participants were financially reimbursed for all costs pertaining to their follow-up visits. In addition, special care was taken to maintain patient comfort level. Health promotional texts were only sent once weekly at convenient times. In days of high security alerts and announced SMS blackouts, the SMS was sent in advance in night to the participant to avoid participant anxiety or uncertainty about continuity of care or reception of services. The data was saved in locked cabinets in the PI office and key was accessible only to limited research staff. Special steps were taken to secure the data on server. The data was completely encrypted thus maintaining patient confidentiality. The information was secured as it was locally hosted. The system was password protected to prevent unauthorized access.

### Pre-testing phase

The first phase of pre-testing of the application (alpha version) was performed by the AKDN eHRC team. All technical glitches within the software were removed. In the second phase (Beta version), the application was tested by the research team. The research team was registered on the software system. Bugs were identified and rectified. This improved version (gamma version) of the application was pretested on actual patients (10% of the total sample size) and the feedback was incorporated. The research team was given two-day training at AKDN eHRC on how to operate the technology and administer the Talking *Rx* software application to the participants. Beside this, AKDN eHRC team was also present on the field to identify any glitches. During this run-in period, participant received the Talking *Rx* Application, glitches were identified and improvements were made. The glitches identified included issues with IVR recordings, scanning of OMR sheet and connection of the software to IVR, Short code issues, and network issues. This final version of the application was deployed during the trial. During the trial, intervention did not undergo any changes in content and deployment frequency.

## Results

**Fig 7:**
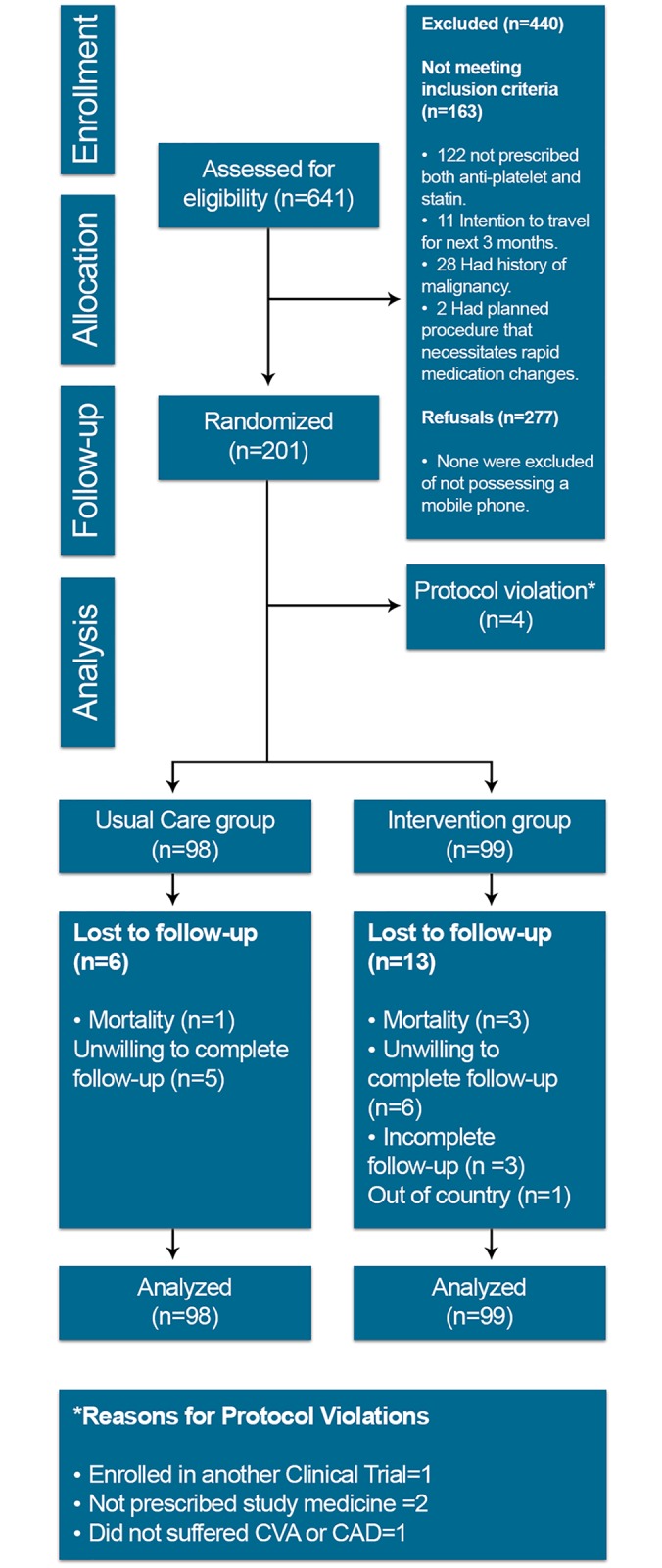
Flow diagram of the study participants.

The Talking Rx study was conducted from April until December 2015. (“Fig 7 Flow Diagram of the Study Participants”) We assessed 641 patients for eligibility, out of which 441 participants were excluded (163 did not match the eligibility criteria and 277 refused to participate in the study). In total 197 participants were randomized into two groups. At the end study, 19 (9.6%) were lost to follow-up.

### Baseline characteristics

A total of 197 participants were analyzed in the study, 99 in intervention group and 98 in the usual care group. The mean age of participants in the intervention and the usual care group was 59.1±11.6 and 57.7±11.1, respectively. Baseline characteristics were equally distributed between the two groups. Table 1

**Table 1:** Baseline characteristics of study participants.

Characteristics	Intervention Group (n = 99) Frequency (%)	Usual Care Group (n = 98) Frequency (%)
**Age in years**^#^ **[Range]**	59.1±11.6 [29–82]	57.7±11.1 [32–84]
**Males**	77(77%)	75(76.53%)
**Years of education**		
Up to 10 years	37(37.37%)	35(35.71%)
Up to 16 years	50(50.50%)	53(54.08%)
Greater than 16 years	12(12.12%)	10(10.20%)
**Family Status**		
Joint Family	50(50.50%)	41(41.83%)
Nuclear Family*	49(49.49%)	57(58.16%)
**Monthly Family income in rupees (Conversion in US** $**)**		
Less than 10,000(<$95)	5(5.05%)	3(3.06%)
10,000–25,000($95-$238)	15(15.15%)	14(14.28%)
26,000–50,000($250-$476)	29(29.29%)	27(27.55%)
51,000–100000($486-$953)	27(27.27%)	31(31.63%)
> than 100000(>$953)	23(23.23%)	23(23.46%)
**Duration of disease (in months)** †	31(6–84)	24 (6–74)
**Disease**		
CVA	50(50.50%)	47(47.95%)
CAD	49(49.49%)	51(52.04%)
**NIHSS Score**† **(National Institute of Health Stroke Severity Scale)**	1(0–2)	1(0–4)
**Hypertension**	63(63.63%)	63(64.28%)
**Diabetes mellitus**	50(50.50%)	55(56.12%)
**Dyslipidemia**	97(97.97%)	91(92.85%)
**Atrial Fibrillation**	1(1.01%)	2(2.04)
**Carotid Stenosis**	2(2.02%)	3(3.06%)
**Coronary Stents placement**	34(34.34%)	27(27.55%)
**CABG**	17(17.17%)	17(17.34%)
**Valve Replacement**	3(3.03%)	3(3.06%)
Central Obesity**		
Male	66(66.66%)	64(65.30%)
Female	20(20.20%)	21(21.42%)
No obesity	13(13.13%)	13(13.26%)
**Cigarette use**		
Smokers	4(4.04%)	10(10.20%)
Non-Smokers	95(95.95%)	88(89.79%)
**History of tobacco chewing**	14(14.14%)	8(8.16%)
**Baseline Mean Morisky Medication adherence**#	6.62± 1.29	6.75± 1.38

^#^ Mean± S.D

^†^ Median (IQR)

^$^ US dollars

*Nuclear family: means living with spouse and children only

** Abdominal obesity, also known as central obesity, is the excessive abdominal fat around the stomach and abdomen has built up to the extent that it is likely to have a negative impact on health. There is a strong correlation between central obesity and cardiovascular disease. It is calculated by measuring individual waist circumference. The cut-offs for waist circumference to report abdominal obesity is different for Asian population and different for both the gender.

### Mean medication adherence

The baseline mean Morisky medication adherence score (MMAS-8) was slightly higher in usual care group 6.77 (SD = 1.36) as compared to 6.68 (SD = 1.28) in the intervention group, though the difference was not statistically significant at baseline. After 3 months of follow-up, the MMAS-8 increased in both groups. While the increase in usual care group was less as compared to intervention group (+0.61 SD = 1.62), there was larger increase in medication adherence score of the intervention group (+0.73 SD = 1.31). The mean difference (95% C.I) between the two group at follow-up was 0.03(S.D 0.13) (95% C.I [-0.23, 0.29]), which was not statistically significant. (*p-Value* = 0.40) Table 2.

**Table 2:** MMAS-8 scores at baseline and 3 months of follow-up among patients randomized to the intervention and usual care groups and MMAS-8 scores at baseline and follow-up among patients with CVA & CAD randomized to the intervention and usual care groups.

	**Intervention group (n = 99)**		**Usual care group (n = 98)**		Mean difference* **(95% C.I)**	**P-value**
	**Baseline**	**3 Months**	**Baseline**	**3 Months**		
All Participants	6.68(1.28)	7.41(0.78)	6.77(1.36)	7.38 (0.99)	0.03 (0.13) (-0.23, 0.29)	0.4
	**Intervention group (n = 50)**		**Usual care group (n = 47)**		Mean difference* **(95% C.I)**	**P-value**
	**Baseline**	**3 Months**	**Baseline**	**3 Months**		
CVA participants only	6.63(1.33)	7.29 (0.82)	6.67(1.37)	7.07(1.24)	0.22(0.22) (-0.21, 0.66)	0.15
	**Intervention group (n = 49)**		**Usual care group (n = 51)**		Mean difference* **(95% C.I)**	**P-value**
	**Baseline**	**3 Months**	**Baseline**	**3 Months**		
CAD Participants only	6.58(1.27)	7.56(0.71)	6.86(1.38)	7.63(0.59)	-0.06(0.13) (-0.39, 0.19)	0.69

*Mean (S.D)

P-value calculated by **Independent t test for two samples**

### Sub-group analysis

We performed a subgroup analysis on CVA and CAD patients. For CVA patients, the baseline mean MMAS-8 was almost similar in both groups in usual care group 6.67(S.D 1.37) as compared to 6.63 (S.D 1.33) in intervention group, the difference was not statistically significant at the baseline. After 3 months of follow-up, the MMAS-8 increased in both groups. While the increase in usual care group was less (+0.4 SD = 1.38), there was larger increase seen in medication adherence score in the intervention group as compared to usual care group (+0.66 SD = 1.32) from baseline. The mean difference (95% C.I) was of 0.22 (SD = 0.22) 95% C.I (-0.20, 0.65) with (*P*-value = 0.15) at the end of follow-up between the two groups was not statistically significant. Table 2. For CAD patients, baseline mean MMAS-8 score was higher in the usual care group 6.86 (S.D 1.38) as compared to 6.58(S.D 1.27) in intervention group, although the difference was not statistically significant at baseline. After 3 months of follow-up, the increase in MMAS-8 was slightly higher in the intervention group (+0.98 SD = 1.26) as compared to usual care group (+0.77 SD = 1.38) from baseline. The mean difference (95% C.I) was of -0.06 (SD = 0.13) (95% C.I = -0.39, 0.19) (p-value = 0.69) at the end of follow-up between the two groups which was not statistically significant tested through Independent t test for two samples.

### Acceptability and sustainability of intervention: End-User feedback via open ended interviews

Open ended interviews of all participants in the intervention was performed at the time of exit interview of the study. All participants were asked three qualitative questions: How did you find the experience of the intervention? What further changes can be made to the intervention? Share your experiences and opinions regarding the intervention? The responses were recorded and summary themes were identified and presented here.

#### Evaluation of program by participants via open ended interviews

All of the participants reported being satisfied with the intervention. They found it an excellent, convenient, useful and informative way of getting knowledge regarding their prescription medicines. Majority felt that the intervention increased their awareness regarding medication usage and side effects. Participants wanted Talking *Rx* to continue for longer than the trial stipulated duration. They also expressed a desire to receive information in a similar format for other medications as well. Participants stated that the SMS reminders helped them remain adherent with their medicines. Most participants found life style modification messages to be beneficial.

#### End-user recommendations and changes suggested

The future recommendation and suggestion included the IVR information to be available in written form as well like a of a text message. Some recommended to reduce the total duration of IVR call and provide information in a more simplified way. Majority of the participants wanted to include information regarding local resources where medicines are easily available. Participants especially stroke patients wanted to receive reminders messages for every dose of their medication. Others suggestions were to include expansion of the intervention beyond two languages (English and Urdu) used in the current study and to add pictorial messages to further improve the understanding of what medications do for those who would learn better through images and videos etc.

#### Uptake of intervention by the participants

The uptake of Talking *Rx* was 84.8%. Table 3 The total number of IVR calls made by the participants in the intervention group was 286 calls. The average duration of IVR recording listened to by the participants was around 8 minutes. The most frequent option accessed by the participants was the correct strength of medicines (27.4%) followed by the frequency of medicine (14.4%). Table 4

**Table 3:** User interface experience.

		Frequency (%)
1	Total No of Participant called	84
2	Uptake of intervention	84(84.84%)
3	Total number of calls made on IVR	286
4	Average number of calls *	2.9± 4.10
5	Average Duration of phone calls in minutes*	8.72± 13.48
6	Average Duration of phone calls in minutes#	4.73[9.9]

*Mean± S.D

^#^ Median [IQR]

**Table 4:** Various IVR options listened to by the participants.

		Frequency (%)
1	Complete information on medicine	36(7.77%)
2	What is dosage (strength) of your medicine	127(27.42%)
3	What is frequency for intake of your medicine	67(14.47%)
4	General information regarding dosage of medicine	53(11.44%)
5	How to properly use your medicine	14(3.02%)
6	How to correctly take your medicine	42(9.07%)
7	What is the purpose of using this medicine?	43(9.28%)
8	What are side effect of your medicine	18(3.88%)
9	What are food-drug interaction	37(7.99%)
10	What are drug-herbal medicine interaction	26(5.61%)

IVR = interactive voice response

## Discussion

The Talking Rx trial is a clinical trial that reports the effectiveness and feasibility of an interactive IT based program to improve medication adherence in stable CVA and CAD patients in a resource challenged setting. The study demonstrated the feasibility of IT based interventions in settings where mobile density and internet coverage is exceptionally excellent despite poverty and other developmental challenges. Participant uptake of the intervention was greater than 80%, there were no exclusions based on technical reasons or lack of mobile phone and mobile coverage In this clinical trial, the intervention did not achieve a statistically significant difference in adherence behavior as measured by the self-reported MMAS-8 scale.

There are several explanations for the observation of the effect on adherence, which improved in the intervention, but failed to achieve statistical significance. Our results may have been affected by a limited duration of follow up; a sustained intervention may have been able to achieve more robust results in adherence behavior. Another explanation for the observed effect is the nature of the population itself, these participants had suffered strokes and heart attacks and received specialist tertiary care, this effected their baseline adherence behavior as compared to populations not exposed to specialist centered care, thus, their baseline adherence was much higher than we anticipated and therefore, the change in medication adherence was low. Most of the participants already had good adherence at baseline as measured by MMAS-8 and therefore, potential for incremental benefit associated with the intervention was small. In future study designs, we would choose participants that report poor baseline adherence and follow them for longer periods of time- upto 6 months. We chose the MMAS-8 as a suitable reporting measure for adherence as this scale has been used in a similar setting previously and it has been translated and validated in Urdu which is the national language of Pakistan. The MMAS-8 was assessed by a research officer in person on follow up visit in person to minimize the effect of social desirability bias that would certainly occur if simple mail in questionnaires were used. However, the social desirability bias is not entirely ruled out in this study and may have biased the results towards minimal effect. It is possible that if more objective measures for measuring intervention effect were chosen e.g. medication refill data or biological measures e.g. low-density lipoprotein cholesterol levels, we may have seen an a larger intervention effect. However, these measures were not logistically feasible in our setting, given non-availability of electronic records with most patients filling their medications from pharmacies in the open market without any standardized documentation, medication refill data could not be obtained. Biochemical tests are costly and impractical in a resource constrained environment and would require repeat hospital visits (which participants cannot afford both due to social and financial reasons) and which would also increase patient-provider interaction and impact the results of the intervention positively as a confounding co—intervention. Electronic pillboxes may potentially provide results that are more accurate. However, a vascular patient’s prescription which includes drugs for multiple risk factors and this creates a challenge capturing actual compliance. Simply opening an electronic pill box registers a dose as administered even if the patient takes only one medicine out of the three that he is prescribed for that time. In future studies, we plan to evaluate the medication adherence through telephonic unannounced pill counts methods, Skype calls or using objective intermediate outcome measures. Additionally, we observed that, the provision of study helpline number to participants in both groups could have affected our study results. Although this help line was not designed as a co-intervention, we believe that it did become a resource for participants enrolled in the usual care group. There was a higher use of help line resources in the control group and this may have resulted in a small-observed difference in medication adherence associated with the intervention in the control group and subsequent decrease in inter-group difference. Lastly, a limitation of this study is that we did not study the effect of intervention on improving patient health related outcomes like decrease in mortality and recurrent strokes or cardiac outcomes that are targets for future larger scale clinical trials.

Although we did not achieve our primary outcome measure, we were able to demonstrate the operational feasibility of using technology driven intervention in LMIC settings. No participant was excluded due to technological reasons and greater than two thirds (84%) used the intervention to learn about their medications. In open-ended interviews, most participants wanted Talking Rx to continue for longer than the trial stipulated duration and found it to be a useful adjunct to the physician visit.

Despite the limitations discussed above, efforts at enabling patients to adhere to medications, which are lifelong, and increasing their prescription knowledge is essential in LMIC settings. In Pakistan, there are about 10 plus brand names for each generic medication which becomes a huge source of prescription error as most medications are accessed over the counter. [27] Drug-dispensing time in the public sector is insufficient for patient instruction, with greater than 3 medications per patient. Despite these regional challenges, the potential to leverage mobile technology to improve chronic disease outcomes is greatly untapped. Pakistan has widespread mobile connectivity, with a cellular density of 77% and more than 137 million mobile users who exchanged 301.7 billion SMS during 2014, a national electronic identification database, low internet and text messaging costs, and availability of 3G and 4G mobile internet. [19]These infrastructure enablers were exploited in TalkingRx.

The major strengths of our study include randomized study design with allocation concealment, blinded outcome assessment, and alignment with standardized guidelines for research in m-health. [14, 28] The content of the intervention was designed by a team of experienced health professionals from both LMIC and developed countries, using evidence based guidelines and grounded in Health Theory. [29, 30] The application was designed by biomedical engineers and programmers working directly with physicians and patient feedback to keep the application simple and user friendly for the community that was to test it. The intervention can be easily replicated in other public hospitals due to the low cost of running it. The technology has the potential for sustainability as it doesn’t require any large-scale capital investments in existing hospital systems and does not require extra personnel or human resources.

## Conclusion

In conclusion, an IVR/SMS based health IT driven was feasible in a busy hospital settings in a resource-constrained setting, although, the intervention did not significantly improve medication adherence it was well received by end users.

## Supporting information

S1 File Participant timeline for Talking Rx_ protocol. (JPG)

S2 File Study Protocol_ERC Approval_Plos one. (DOC)

S3 File DownloadReceipt. (PDF)

S4 File CONSORT 2010 checklist. (DOC)
