# Supplementary figures and images for: Making prescriptions “talk” to stroke and heart attack survivors to improve adherence: Results of a randomized clinical trial (The Talking Rx Study)
Source: PLoS One. 2018 Dec 20;13(12):e0197671. doi: 10.1371/journal.pone.0197671 (PMC6301764; doi:10.1371/journal.pone.0197671)

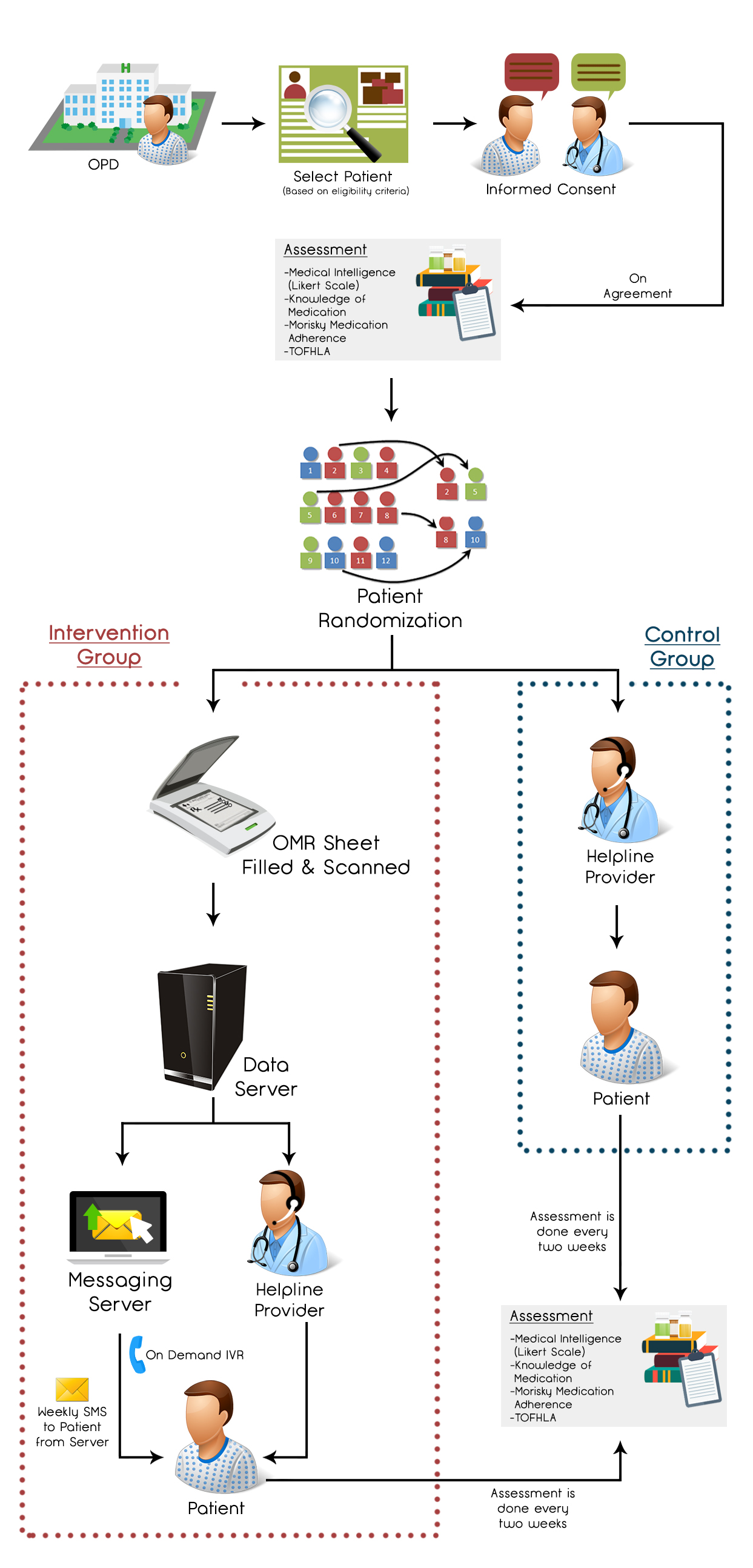

Supplement: S1 File — (JPG) [file pone.0197671.s001.jpg]
