# Supplementary material for: Making prescriptions “talk” to stroke and heart attack survivors to improve adherence: Results of a randomized clinical trial (The Talking Rx Study)
Source: PLoS One. 2018 Dec 20;13(12):e0197671. doi: 10.1371/journal.pone.0197671 (PMC6301764; doi:10.1371/journal.pone.0197671)
Supplement: S2 File — (DOC) [file pone.0197671.s002.doc]

**Using a Tailored Health Information Technology Driven Intervention to Improve Health Literacy and Medication Adherence in a Pakistani Population with Vascular Disease**

Ayeesha Kamal^1^ , Saleem Sayani^2^ Saleem Virani^3^, Saira Bokhari^4^.

^1^Stroke Services, Section of Neurology, Dept. of Medicine, Aga Khan University

^2^E- Health Resource Center, Aga Khan University

^3^Baylor College of Medicine, Dept. of Medicine: Athero and Lipo

^4^ Section of Cardiology, Dept. of Medicine, Aga Khan University

CONTENT

|  | **Page no.** |
| --- | --- |
| 1.0 Background | 03 |
| 2.0 Hypothesis | 03 |
| *2.1. Health Literacy* | 03 |
| *2.2. Medication adherence* | 04 |
| 3.0 Aims of the Study | 04 |
| 4.0 Methodology | 04 |
| *4.1. Study Design* | 04 |
| *4.2. Study Setting* | 04 |
| *4.3. Sample* | 04 |
| *4.4. Inclusion Criteria* | 04 |
| *4.5. Exclusion Criteria* | 05 |
| *4.6. Data Collection* | 05 |
| *4.7. Recruitment Plan* | 05 |
| *4.8. Pre-intervention* | 05 |
| *4.9. Randomization* | 05 |
| *4.10. Control Group* | 05 |
| *4.11. Intervention* | 05 |
| 5.0 Outcomes | 06 |
| 6.0 Data Analysis | 06 |
| 7.0 Ethical Considerations | 06 |

**1.0 BACKGROUND**

Vascular risk ultimately manifested as myocardial infarction and stroke contributes to about 3.87 million premature deaths in the 30 - 69 year old age group in Pakistan. Stressed on the fact that, addressing the above mentioned risk factors as strategic, can reduce this premature death toll by 20% in Pakistan Cardiovascular Disease (CVD), remains a major cause of morbidity and mortality in patients from South East Asia. This region comprises of India, Pakistan, Bangladesh, Nepal, and Sri Lanka. Collectively, these countries represent around one-fifth of the global population. In one of the largest case-control studies of 15,152 cases of first myocardial infarction (MI) and 14,820 controls, it was shown that South Asian participants had one of the lowest age at the time of their MI (mean = 53 years) compared with 63 years for participants from China or Western Europe. Nearly 10% of these cases in South Asian participants occurred in those aged 40 or below. These data indicate a growing epidemic of premature CVD in South Asian populations.

Management of CVD factors has seen tremendous improvements in the Western countries with greater than 90% of the patients discharged on medications known to reduce incidence of recurrent cardiovascular disease in these patients (e.g., aspirin, statins). Although most patients admitted with acute coronary syndrome or acute ischemic stroke in South Asian countries receive these evidence-based treatments, their overall continuation in the outpatient phase of care remains low. Patient from Pakistan are uniquely challenged in this respect because the overall literacy rates remain one of the lowest in Pakistan among South Asian Countries. In addition, a great majority of Pakistani patients often do not understand or follow health prescriptions (which are still written in English). Additionally, due to an unregulated health industry, they frequently take multiple opinions and prescriptions from different physicians. This lack of understanding of why they are taking their medication leads a reduced adherence to these life-saving medications and increases their risk for drug-drug interactions and serious adverse reactions.

The purpose of this study is to develop and pilot test a tailored health information technology driven intervention in Pakistani patients with coronary heart disease and ischemic strokes receiving care in outpatient setting in the Aga Khan University Hospitals in Karachi, Pakistan.

**2.0 HYPOTHESIS**

**2.1 Health literacy:**

**Null Hypothesis:** there will be no difference in health literacy of patients in the intervention and control groups.

**Alternative Hypothesis:** the health literacy score of patients receiving IT driven health information intervention will be higher than patients receiving conventional health education.

**2.2 Medication Adherence:**

Null Hypothesis: There will be no difference on the medication adherence levels of the patients in the intervention and control group.

Alternative Hypothesis: The medication adherence level will be higher among the patients receiving IT driven health information intervention, as compared to the patients receiving conventional health education in the clinics.

**3.0 AIMS OF THE STUDY**

Aim 1: To develop and pilot test a health IT driven intervention delivered in local language (Urdu) which has two components: a. Tailored text messages or voice messages regarding indications, instructions, frequency of intake of statins and antiplatelet medications b. Weekly text reminders to patients regarding statins and antiplatelet medications.

Aim 2: To perform a pilot intervention among 200 patients with coronary heart disease (CHD) or ischemic strokes to determine whether this tailored intervention is associated with an improvement in health literacy and medication adherence compared with usual care at 3 months of follow-up.

Aim 3: To perform a summative evaluation by a group of end users to further improve this intervention for future large scale studies in cardiovascular disease patients.

**4.0 METHODOLOGY**

**4.1 Study Design:**

The study will be a randomized controlled trial (RCT) with blinded outcome assessment.

**4.2 Study Setting**

The study will be conducted at the out-patient cardiology and neurology clinics of The Aga Khan University Hospital, Karachi.

**4.3 Sample**

Following an inclusion criteria, a sample of 200 patients (100 in each arm) will be recruited in the study via a method of convenience sampling.

**4.4 Inclusion Criteria**

Patients above 18 years old, diagnosed with either stroke or coronary artery disease diagnosis at least 1 month back, have a personal mobile phone, can send and receive messages in Urdu / English. Disability score should be <3. Do not intend to travel for the next 3 months. Has been prescribed both anti-platelet and a statin.

**4.5 Exclusion Criteria**

Excluded if suffering from any malignancy.

**4.6 Data Collection**

Data on the socio-demographic status of the patients will be collected through a socio-demographic checklist. Similarly, health literacy will be assessed via Test of Functional Health Literacy in Adults (TOFLHA). Likewise, the medication adherence will be assessed using the Morisky medication Adherence scale.

**4.7 Recruitment Plan**

Patients will be recruited from the outpatient cardiology and neurology clinics of AKUH. After assessing for the eligibility, patients will be approached for their participation in the study. After explaining the purpose and methods, if they consent to participate in the study, they will be recruited in the study.

**4.8 Pre-intervention**

At the time of enrollment in the study (before randomization), the baseline data on the socio-demography, TOFLHA and Morisky medication adherence scale will be obtained.

**4.9 Randomization**

After obtaining the baseline information, patients will be randomized in to either intervention or control group through central randomization process by clinical trial unit.

**4.10 Control Group**

The control group receives conventional health education by nurses and their physicians, after their out-patient visit.

**4.11 Intervention**

1. Physicians seeing patients in the outpatient clinics will write patient’s statin and/or antiplatelet prescription and patient’s cell phone numbers on an OMR optical mark recognition (OMR) sheet. The sheet will also have dosage, route, frequency, and duration options for respective medications. The physician will color the appropriate specification for a medication instead of writing the prescription by hands.
2. The research officer would then scan the prescription sheet and upload the scanned copy to the database server.
3. Software in the server will analyze the prescription and generate the following two outputs for each medication:
   1. Output 1 (basic drug prescription information): Name of the medication, dosage, route, frequency, number of days, any special instruction, and Short Message Service (SMS ) and Interactive Voice Response (IVR) code.
   2. Output 2 (detailed drug information): Indication, contraindication, side effects, adverse effects, drug interaction and food interaction.
4. SMS software would then send a voice and text message to the patient’s cell phone with output 1. The voice message will be in Urdu. The prescription would be read to the patient through voice message in Urdu. The text message with output 1 would be sent in Roman Urdu.
5. Output 1 will also generate a unique code for each patient. The patient will use this code, at any time of his/her convenience, to request for a repeated text or voice message for output 1 or to request a text or voice message for output 2 (if the patient is interested in receiving detailed drug information). The patient would be able to receive both the outputs multiple times, on-demand.
6. Patient will also receive a weekly text message reminding them to take their statin and/or antiplatelet medication.

**5.0 Outcomes**

The outcome will be assessed at three months of delivering the intervention, through the same instruments used before the intervention.

The outcomes of the study will be health literacy and medication adherence levels.

**6.0 Data Analysis**

Descriptive Statistics: Mean, Median frequencies/percentages will be calculated for all the descriptive data.

Inferential Statistics: t-test for two independent samples will be applied to evaluate the difference in the mean health literacy levels of both the groups.

**7.0 Ethical Considerations**

The study will be commenced after receiving an approval from the institutions ethics review committee (ERC). Written and verbal consent from the participants will also be obtained. Confidentiality and anonymity of the participants will be maintained by providing a study ID number. Patients will also be assured that their responses will be confidential and anonymous because of the allocation of study ID. They will be informed about their right to withdraw from the study any time, and they will be reassured that withdrawing from the study will not in any way affect their treatment plan. All the data will be kept in the locked cabinets and the information on the computer will be secured by password. In order to maintain the privacy of the patient, the patients will be approached in a private room by the research assistant.
