# Supplementary material for: Making prescriptions “talk” to stroke and heart attack survivors to improve adherence: Results of a randomized clinical trial (The Talking Rx Study)
Source: PLoS One. 2018 Dec 20;13(12):e0197671. doi: 10.1371/journal.pone.0197671 (PMC6301764; doi:10.1371/journal.pone.0197671)
Supplement: S3 File — (PDF) [file pone.0197671.s003.pdf]

ClinicalTrials.gov Protocol Registration and Results System (PRS) Receipt
Release Date: December 12, 2017

ClinicalTrials.gov ID: NCT02354040

Study Identification

Unique Protocol ID: 3165- MED-ERC_14

Brief Title: Using a Tailored Health Information Technology Driven Intervention to Improve Health Literacy and Medication Adherence (TalkingRx)

Official Title: Using a Tailored Health Information Technology Driven Intervention to Improve Health Literacy and Medication Adherence in a Pakistani Population With Vascular Disease

Secondary IDs:

Study Status

Record Verification: December 2017

Overall Status: Completed

Study Start: April 2015 [Actual]

Primary Completion: December 2015 [Actual]

Study Completion: December 2015 [Actual]

Sponsor/Collaborators

Sponsor: Aga Khan University

Responsible Party: Principal Investigator
Investigator: Dr. Ayeesha Kamran Kamal [akamal]
Official Title: Ayeesha Kamran Kamal
Affiliation: Aga Khan University

Collaborators: Baylor College of Medicine

Oversight

U.S. FDA-regulated Drug:

U.S. FDA-regulated Device:

U.S. FDA IND/IDE: No

Human Subjects Review: Board Status: Approved
Approval Number: 3165- MED-ERC_14
Board Name: Pakistan: Research Ethics Committee
Board Affiliation: Aga Khan University Hospital
Phone: 00922134864880
Email: gulshan.kalani@aku.edu
Address:

Research Office 1st Floor Juma Building
Aga Khan University
Stadium Road
Karachi 74800
Pakistan

Data Monitoring: No

FDA Regulated Intervention: No

Study Description

Brief Summary: Although most patients admitted with acute coronary syndrome or acute ischemic stroke in South Asian countries receive these evidence-based treatments, their overall continuation in the outpatient phase of care remains low. Patient from Pakistan are uniquely challenged in this respect because the overall literacy rates remain one of the lowest in Pakistan among South Asian Countries. In addition, a great majority of Pakistani patients often do not understand or follow health prescriptions (which are still written in English). Additionally, due to an unregulated health industry, they frequently take multiple opinions and prescriptions from different physicians. The investigators propose to develop a “talking prescription” for patients with stroke or myocardial infarction for secondary prevention. This will enable them to understand their medications better, improve health literacy and adherence. This is an IT enabled health literacy intervention. Physicians will prescribe statin and/or antiplatelet to the selected patients and enter the necessary details on an Optical Mark Recognition (OMR) sheet.Patients will be assigned to either of the 2 arms--either regular care or talking prescriptions. Follow-up will be done at 3 months post recruitment for behavioral knowledge assessment and adherence assessment.

Detailed Description: Physicians seeing patients in the outpatient clinics will write patient’s statin and/or antiplatelet prescription and the patient’s cell phone numbers on an Optical Mark Recognition (OMR) sheet. The sheet will also have dosage, route, frequency and durations options for respective medications. The physician will color the appropriate specification for a medication instead of writing the prescription by hand. Statin medications include lovastatin, fluvastatin, simvastatin, atorvastatin, pravastatin, rosuvastatin or pitavastatin. Antiplatelet medications include aspirin, clopidogrel, aspirin plus dipyridamole, cilostazol and prasugrel.

The unit receptionist would then scan the prescription sheet and upload the scanned copy to the database server.

Software in the server will analyze the prescription and generate the following outputs for each medication:

Output 1 (basic drug prescription information) (Option 1): Name of the medication, dosage, route, frequency, number of days, any special instruction, and Short Message Service (SMS) and Interactive Voice Response (IVR) code.

Output 2 (detailed drug information):

Indication (Option 2), Contraindications and side/adverse effects (Option 3), and Drug-drug interactions and drug-food interactions (Option 4).

SMS software would then send a voice and text message to the patient’s cell phone with Output 1. The voice message will be in Urdu. The prescription would be read to the patient through voice message in Urdu. The text message with Output 1 would be sent in Roman Urdu.

Output 1 will also generate a unique code for each patient. The patient will use this code, at any time of his/her convenience, to request for a repeated text or voice message for output 1 or to request for a repeated text or voice message for Output 2 (if the patient is interested in receiving detailed drug information). The patient would be able to receive both the outputs multiple times, on-demand.

Patient will also receive a weekly text message reminding them to take their statin and/or antiplatelet medication.

Conditions

Conditions: Vascular Disease
Cerebrovascular Disorders
Ischemic Heart Disease
Coronary Artery Disease

Keywords: Stroke, Myocardial Infarction, Adherence, Health Literacy, Implementation, Developing Country

Study Design

Study Type: Interventional

Primary Purpose: Health Services Research

Study Phase: N/A

Interventional Study Model: Parallel Assignment

Number of Arms: 2

Masking: Single (Outcomes Assessor)

Allocation: Randomized

Enrollment: 200 [Actual]

Arms and Interventions

Arms	Assigned Interventions
Experimental: Talking Rx<br>Assigned to receive Health Literacy and Reminder Updates via the IT based intervention Talking Rx, in addition to Usual Care for patients in the intervention group. The physician written prescription for anti-platelet and statin will be transferred on an OMR sheet and will be scanned. The information on the prescription (dose, name of the medication, duration, route or any other special instruction) will be sent to the patients via a text and a voice SMS (in Urdu language). The patients also receive an individualized code that helps them request for repeated reminders for their medication timings. However, a weekly medication reminder SMS will be sent to the patients in the intervention arm.	Behavioral: Talking Rx<br>For patients in the intervention group, the physician written prescription for anti-platelet and statin will be transferred on an OMR sheet and will be scanned. The information on the prescription (dose, name of the medication, duration, route or any other special instruction) will be sent to the patients via a text and a voice SMS (in Urdu language). The patients also receive an individualized code that helps them request for repeated reminders for their medication timings. However, a weekly medication reminder SMS will be sent to the patients in the intervention arm.
No Intervention: Usual Care, Prescriptions and Counselling	

Arms	Assigned Interventions
Assigned to receive a standard prescription and Counselling only, there are no cointerventions in this group	

Outcome Measures

Primary Outcome Measure:

1. Medication Adherence
   Morisky Medication Adherence Self Reported Scale

   [Time Frame: 3 Months]

Secondary Outcome Measure:

2. Health Literacy
   Adapted and Modified TOFHLA Scale - Test of Health Literacy in Adults

   [Time Frame: 3 Months]

Eligibility

Minimum Age: 18 Years

Maximum Age:

Sex: All

Gender Based:

Accepts Healthy Volunteers: No

Criteria: Inclusion Criteria:

- Adult Men and Women ,18 years old
- Ischemic Stroke or CAD which is stable and outpatient based
- Own Cell Phone
- Can read local language,hear Cell phone,See a short text message
- Willing to Give Written Informed Consent
- Consent to follow up
- Stroke and CAD are stable specifically, not requiring medical procedures that would necessitate frequent interruptions in medications.
- Modified Rankin Score (MRS) Disability score should be less than 2.

Exclusion Criteria:

- No Ischemic Stroke or CAD
- Ischemic Stroke and CAD are unstable e.g. Unstable Angina, Acute MI, Need for CABG, Need for CEA, Need to rapidly adjust medication and inpatient care
- Biological impairment in reading or responding to short text messages such as (but not limited to) loss of vision, visual field cuts, aphasia.
- Diagnosed organ dysfunction or malignancy requiring stopping or adjustment of medications
- Plans to travel outside the country inside the two months following enrollment
- Known allergy or adverse reaction.
- Absolute or relative contraindication to antiplatelet or statin e.g chronic liver disease, myopathy, Thrombotic Thrombocytopenic Purpura, GI bleed , Active Gastritis

Contacts/Locations

Central Contact Person: Salim Virani, MD
Telephone: 7137985846 Ext. 5846
Email: virani@bcm.edu

Central Contact Backup: Ayeesha K Kamal, MBBS
Telephone: 9221 34930051 Ext. 4559
Email: ayeesha.kamal@aku.edu

Study Officials:

Locations: Pakistan
Aga Khan University, Clinical Trial Unit
Karachi, Pakistan

IPDSharing

Plan to Share IPD:

References

Citations:

Links:

Available IPD/Information:

U.S. National Library of Medicine | U.S. National Institutes of Health | U.S. Department of Health & Human Services
